# Predation Risk Does Not Delay Breeding but Reduces Nest Survival in High‐Arctic Shorebirds

**DOI:** 10.1002/ece3.72997

**Published:** 2026-03-18

**Authors:** Laura Bonnefond, David Pinaud, Loïc Bollache, Thomas Pagnon, Eric Buchel, Brigitte Sabard, Vladimir Gilg, Jérôme Moreau, Olivier Gilg

**Affiliations:** ^1^ Centre d'Etudes Biologiques de Chizé (UMR 7372), CNRS & La Rochelle Université Villiers‐en‐bois France; ^2^ Université de Bourgogne Europe Dijon France; ^3^ Université Marie et Louis Pasteur, CNRS, Chrono‐Environnement (UMR 6249) Besançon France; ^4^ Groupe de Recherche en Ecologie Arctique (GREA) Francheville France; ^5^ UMR CNRS 6282 Biogéosciences, Équipe Écologie Évolutive, Université de Bourgogne Europe Dijon France

**Keywords:** *Calidris alba*, *Calidris alpina arctica*, GPS telemetry, nest initiation, predator–prey interactions, *Vulpes lagopus*

## Abstract

Understanding predator–prey interactions is crucial for correctly answering many fundamental research questions, given their behavioural, ecological and evolutionary consequences. Such interactions can result in the direct consumption of the prey, but also in behavioural responses such as changes in breeding phenology. Space use of predators and the associated predation risk is a dynamic process, resulting in constant adjustments by the prey at different spatiotemporal scales. In the Arctic, predator–prey interactions are expected to change owing to the ongoing climate change and the associated shifts in ecosystem composition. However, most studies investigating the impact of predation on ground‐nesting birds use data from artificial nests that lack the behavioural component of incubation. Consequently, studies investigating the impact of predation risk on real nests are needed, especially in arctic ecosystems of relatively low productivity. Using nest data collected over 3 years in Northeast Greenland, we investigated how spatial variations in predation risk by arctic foxes ( *Vulpes lagopus* ) impacted daily nest survival rates and start of incubation dates of two sympatric species of sandpipers: the sanderling ( *Calidris alba* ) and the dunlin ( *Calidris alpina arctica* ). Fox spatial activity was estimated by calculating their Autocorrelated kernel Utilisation Distributions based on fine‐scale GPS data. We found that both sandpiper species had higher daily nest survival rates in areas of low fox activity. However, sandpipers initiated their nests earlier in areas of high fox activity. Together, these results show that predation risk does not delay the breeding phenology of high‐Arctic sandpipers, and that predation risk clearly translates into lower nest survival, as often claimed by previous studies using artificial nests.

## Graphical abstract

We assessed the impact of arctic foxes' predation pressure on two sympatric sandpiper species. Fox activity influenced the breeding ecology of both sandpipers, with earlier nest initiations and lower nest survival rates in areas of high fox activity.

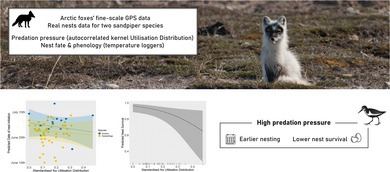

## 1 Introduction

Interspecific interactions are widespread in natural ecosystems (Thompson 1988) and have the potential to drive population dynamics and ecosystems composition (Aschehoug et al. 2016; Strauss and Irwin 2004; Svenning et al. 2014). Among these, predator–prey interactions are perhaps one of the most important in an evolutionary context since they can influence the behaviour (Abrams 2000), demography (Gese and Knowlton 2001), distribution (Wisz et al. 2013), physiology (Hawlena and Schmitz 2010), morphology (Kishida et al. 2010), and activity patterns (Monterroso et al. 2013) of both predator and prey. Predator–prey interactions have been studied by ecologists for decades (Abrams 2000; Sih 1984), but we still do not fully understand their implications and consequences in natural environments (Guiden et al. 2019).

Nest predation is a major cause of breeding failure in birds (Lima 2009) and has important consequences at both the individual and population levels (Ibáñez‐Álamo et al. 2015; Martin 1995). Nest predation rates are not homogenous in space and time (e.g., Lecomte et al. 2008; McKinnon et al. 2010), and birds must thereby adjust their decisions to mitigate predation risk. For instance, they can select nesting habitats where predators are less abundant (Clermont, Woodward‐Gagné, and Berteaux 2021; Flemming, Nol, Kennedy, and Smith 2019; Johnson‐Bice et al. 2024), difficult for predators to access (Anderson et al. 2015; Clermont, Grenier‐Potvin, et al. 2021) and detect (Colombelli‐Négrel and Kleindorfer 2009), or sites with an open view that favour early detection of predators (Cunningham et al. 2016; Dorsey et al. 2025; Korne et al. 2020). Furthermore, the presence of alternative resources for predators can either increase (apparent competition, Beardsell et al. 2023; Dulude‐de Broin et al. 2023; Flemming, Nol, Kennedy, Bédard, et al. 2019) or decrease (apparent mutualism; Beardsell et al. 2022; Pedersen et al. 2018) nest predation risk. Finally, predators can also influence the behaviour of breeding birds by impacting their breeding phenology. For example, studies have shown that birds nesting in risky areas can initiate their nests later (Byrkjedal 1980; Harts et al. 2016; but see Fontaine and Martin 2006; Smith and Wilson 2010), either to benefit from a “dilution effect” (i.e., more prey being available to predators later in the season, Gorosito et al. 2024; Reneerkens et al. 2016), or simply because the best and safer territories are already occupied by early breeding (and likely more competitive) congeners (Currie et al. 2000; Fontaine and Martin 2006).

To date, most research has focused on the impact of predators on their main prey (e.g., Salo et al. 2010; Wirsing et al. 2021). However, recent studies have shown that incidental (or “accidental”) prey (i.e., species that are not actively searched for by a predator, but consumed opportunistically, Cornell 1976) can also be strongly impacted by predation risk, both through consumption of the nest or because of predator‐induced behavioural changes such as a shift in habitat selection patterns (Clermont, Grenier‐Potvin, et al. 2021; Vickery et al. 1992; Vigallon and Marzluff 2005). Due to their small size and low breeding densities (hence with limited benefit for the predators that generally prefer larger and more abundant prey), sandpipers (*Charadriidae* family) are usually considered incidental prey on their Arctic breeding grounds for most predators (Flemming, Nol, Kennedy, Bédard, et al. 2019; McKinnon et al. 2014). However, in such an ecosystem, predation on their nests can be extremely high (e.g., up to 96% of all nests predated, Flemming, Nol, Kennedy, Bédard, et al. 2019) and has the potential to drive a decline in shorebirds' populations (Blomqvist et al. 2002). Therefore, different shorebird species have evolved different strategies to mitigate predation risk: some display highly‐cryptic plumage (Ekanayake et al. 2015), show predator‐deception mechanisms (e.g., “broken wing behaviour”, De Framond et al. 2022) or flush far from the nest (Smith and Edwards 2018). Some species can also select specific types of vegetation or land cover for nesting to mitigate predation risk, whereas others cannot because of ecological constraints (Léandri‐Breton and Bêty 2020). Shorebird species also differ in terms of breeding strategy, with nests of uniparental individuals (i.e., only one parent incubating) being generally more prone to predation compared with biparental nests (i.e., the two parents share the incubation, Smith et al. 2012; Smith and Wilson 2010), probably owing to a difference in terms of time spent off the nest (Meyer et al. 2020) and movements during incubation (Smith et al. 2012).

Recent studies have investigated the impact of predation risk on Arctic‐breeding shorebirds and emphasised a strong potential for predators to impact nest fate and phenology (Dulude‐de Broin et al. 2023; Flemming, Nol, Kennedy, Bédard, et al. 2019; Léandri‐Breton and Bêty 2020; Smith et al. 2010). However, most studies have been conducted in relatively rich polar environments, where predators can benefit from abundant, spatially predictable food sources (e.g., nesting geese, Clermont, Grenier‐Potvin, et al. 2021; Flemming, Nol, Kennedy, Bédard, et al. 2019; Flemming et al. 2016). Hence, studies conducted in these rich Arctic ecosystems do not generally allow for extrapolations since predation pressure patterns change when resources become less abundant (Beardsell et al. 2022; Pedersen et al. 2018). Furthermore, most researches conducted in the Arctic use artificial nests to quantify the impact of predation on nest survival (e.g., Bentzen et al. 2017; Brown et al. 2022; Flemming, Nol, Kennedy, Bédard, et al. 2019; Lamarre et al. 2017; Léandri‐Breton and Bêty 2020; but see McKinnon et al. 2014; Smith and Wilson 2010), a method that can be biased, for example, if nest density or behaviour of incubating birds influences predation rates (Meyer et al. 2020; Smith et al. 2012; Smith and Wilson 2010). We thereby have limited knowledge on how predators impact nest survival rates and sandpipers’ breeding behaviour in a natural context within relatively poor Arctic terrestrial ecosystems (i.e., with variable but overall low prey abundance, which represent a significant part of the global Arctic ecosystem).

This study aimed at investigating the impact of predation on the breeding behaviour of incidental preys in a relatively poor arctic ecosystem, inferred from real nests data. Using arctic foxes ( *Vulpes lagopus* ) and two species of sandpipers as model species, we hypothesised that daily nest survival rates and breeding phenology would be influenced by predator activity, and that the two bird species would respond differently to predation pressure, owing to their species‐specific ecology. We predicted that (1) nests would be initiated later in riskier areas (to benefit from a “dilution effect” or due to early season competition for the best and safest territories; Currie et al. 2000; Fontaine and Martin 2006), (2) daily survival rates (DSR) of sandpiper nests would be lower in areas of high predator activity and (3) different bird species would exhibit different responses to predation pressure (i.e., in terms of nesting phenology and DSR).

## 2 Materials and Methods

### 2.1 Study Area and Species

**FIGURE 1:**
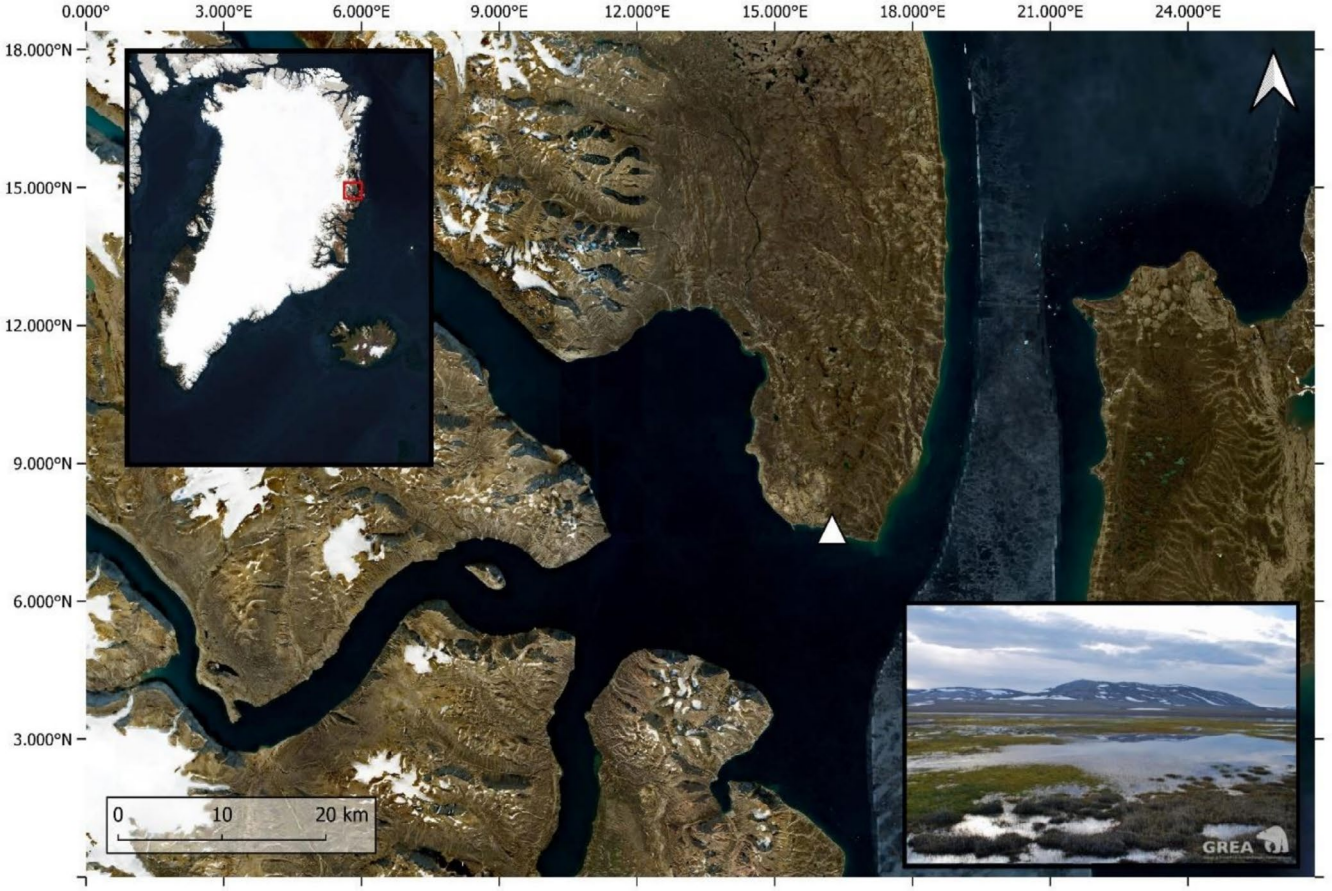
Map of the study area. The red square indicates the location of the study area in Northeast Greenland. The white triangle represents the centre of the prospected study area. The photograph shows a typical landscape of the location. © MapTiler and OpenStreetMap contributors, 2021 (map) and Vadim Heuacker (photograph).

Data were collected at Hochstetter Forland (75.15° N, 19.70° W), a 18 km^2^ flat lowland with numerous lakes and ponds in Northeast Greenland (Figure 1; Meltofte et al. 1981) belonging to the shrub tundra subzone biome and characterised by graminoids, prostrate dwarf‐shrubs and forb tundra on moist soils, and prostrate dwarf‐shrubs tundra on dry soils (Walker et al. 2005).

Many shorebird species, including sanderlings ( *Calidris alba* ) and dunlins ( *Calidris alpina arctica* ), commonly migrate to and breed in Northeast Greenland during the Arctic summer (Boertman 1994; Lyngs 2003). Both species typically arrive just before snowmelt and start laying their eggs in June (Meltofte 2007). Like most sandpipers (genus *Calidris*), clutch size of sanderlings and dunlins is usually four eggs (MacLean 1972), with an incubation period of ca. 22 days (Liebezeit et al. 2007; Mabee et al. 2006; Reneerkens et al. 2011). The two species nest directly on the ground, but dunlins generally select moister habitats to establish their nests (Cunningham et al. 2016), while sanderlings preferentially select drier habitats and avoid wet areas (Pellissier et al. 2013). They also differ in their breeding strategy: although both parents provide parental care in dunlins (i.e., biparental care), sanderlings exhibit a mixed strategy meaning that they can be either biparental, uniparental (care is provided by one parent only), or even switch from biparental to uniparental during the breeding season (Etchart et al. 2025; Meyer et al. 2020). Nest predators in our study area include arctic foxes, stoats ( *Mustela erminea* ), ravens ( *Corvus corax* ), snowy owls ( *Bubo scandiacus* ), skuas (*Stercorarius* spp.), and gulls (*Larinae* spp.).

The arctic fox is the main terrestrial predator in the region (Gilg et al. 2006; Meltofte et al. 1981), although their density is relatively low (Norén et al. 2011; unpublished data). Although foxes preferentially prey on collared lemmings ( *Dicrostonyx groenlandicus* ) when available, they still have a relatively broad diet made of waterfowls, eggs, passerines, and carcasses of large mammals (Gagnon and Berteaux 2009; Gilg et al. 2006; Schmidt et al. 2022; Ungar et al. 2021). Lemming densities at Hochstetter are relatively stable and low (ranging between 0.25 and 1.08 lemming/ha during the 3 years for which fox data were used; see below) compared to other Arctic sites (Ehrich et al. 2020). In response, arctic foxes rely more on alternative prey in our study area (Schmidt et al. 2022) even if resource abundance is relatively low and does not generally greatly vary from year to year. During the Arctic summer, and particularly in years when food is abundant, arctic foxes can produce large litters (Fuglei and Ims 2008), which increases their nutritional needs. To take advantage of abundant but time‐limited and pulsed resources, arctic foxes are capable of adjusting their movements and foraging behaviour at the intraseasonal scale (Bonnefond et al. 2025). In Greenland, the arctic foxes' breeding season coincides with those of birds, including shorebirds. These shorebirds can then constitute a significant nutrient intake for arctic foxes (Mckinnon, Berteaux, et al. 2013; McKinnon, Nol, and Juillet 2013; Schmidt et al. 2022).

### 2.2 Fox Data

Arctic foxes were captured between 2017 and 2021 (with the exception of year 2019) using Tomahawk cage traps or padded leg‐hold traps (model Victor No. 1 Soft‐Catch, Oneida Victor Ltd., USA). Handling and tagging of arctic foxes was allowed by the Government of Greenland, Ministry of Domestic Affairs, Nature and Environment‐NNPAN (permits C‐17‐3‐28, C‐20‐3‐19, C‐21‐3‐22). We did not have to anaesthetize the arctic foxes, which were always very tame (see also Warret Rodrigues and Roth 2023). Handling and tagging (by two trained persons) were kept as short as possible (i.e., 20–30 min), with fox eyes simply covered by an opened sock (to allow normal breathing). We removed data from 2018 (*N = 1* fox) since foxes' spatial behaviour was very unusual during this year, and bird nests were very rare, owing to extreme snow conditions (Schmidt et al. 2019). Foxes were fitted with GPS collars powered by a solar panel, allowing remote data download via UHF transmission (“RadioTag‐14” from Milsar Ltd., Nicosia, Cyprus, and “Felis” from Ecotone Telemetry, Gdynia, Poland; Milsar Base Station with UHF download [2.4 GHz ISM Band]). All collars weighed between 100 and 120 g, that is, less than 3.5% of the body mass of foxes. All individuals were adults when captured and sex was determined prior to release. The collars were set to send a GPS position every 1–5 min in summer, with a fix‐rate schedule varying between individuals. To homogeneously estimate movement parameters between individuals, we resampled our data to keep only one position every 4 min for each individual, as recommended by Poulin et al. (2021). We discarded GPS positions with dilution of precision values ≥ 10 and unrealistic speeds (i.e., > 20 km/h between successive positions; Pletenev et al. 2021). We also removed the first 2 days of data following capture to avoid including movements that could be influenced by capture‐related stress (Clermont, Grenier‐Potvin, et al. 2021).

We only kept fox data collected during the sandpipers' incubation period for each individual year. This period started with the initiation of the first sandpiper nest of the season and ended with the hatching date of the last breeding sandpiper for the given year (Table S1.1). The date of first nest initiation varied between years and study areas but was comprised between the 10th of June (2021) and the 26th of June (2017). Similarly, the date of last hatching was between the 27th of July (2021) and the 6th of August (2017; Table S1.1). This left us with a total of 22,220 fox GPS positions (mean ± SD per fox‐year: 4444 ± 5373; range 1140–13,873; Table S1.2) collected from 4 individuals (2 females, 2 males). Since one male was tracked for two successive years, we ended up with 5 fox‐years of data (Table S1.2).

### 2.3 Sandpiper Nest Data

The protocol for searching, handling, and monitoring sandpipers (i.e., dunlin and sanderling) nests was similar in all years: sandpiper nests were searched for by foot by experienced field biologists (team size of six persons in each year) in the same study area from 2017 to 2021 (Table S1.1). Since no fox was tracked in the area in 2019, we did not consider this year in the subsequent analyses. We also removed year 2018 from our analyses (*N = 4* nests) since this year was biologically very different from the three other ones (see above). The laying and hatching dates were estimated by floating two to three eggs in each clutch (Liebezeit et al. 2007), and the nests were visited again 2–3 days prior to the expected hatching date to search for early signs of hatching (e.g., starred or pipped eggs). All but a few nests (*N = 10* nests not equipped) were monitored with temperature loggers (TinyTag Plus2 TGP‐4020; Gemini Data Loggers Inc., West Sussex, UK) that allowed us to document the parent(s) incubation behaviour (i.e., number and duration of nest recesses). The temperature loggers recorded temperature data at a one‐minute interval for 22 days. The nest fate (i.e., the state of the nest at the end of the incubation, either hatched or predated) was inferred from temperature patterns extracted from the loggers which leave a particular signature depending on the fate (Meyer et al. 2020; Moreau et al. 2018). The use of small temperature loggers has proven effective in determining nest fate in shorebirds (Weidinger 2006) while minimising nest disturbance, thereby resulting in no negative impact on nest survival (Mougeot et al. 2014; Sutti and Strong 2014). A total of 68 nests were discovered during the study period. We regularly revisited the few nests that were not equipped with temperature loggers (i.e., every 1–5 days) until signs of hatching or predation were found. If at least one chick hatched, the nest was considered successful. A nest was considered predated if the temperature loggers recorded a characteristic temperature profile (i.e., a sudden and rapid drop in temperature, Meyer et al. 2020) or, for the 10 nests that had no temperature logger, if signs of depredation were found upon visit (e.g., empty nest with chewed egg shells and/or fox scent or scat in the nest cup; Green et al. 1987). The predator's identity could not always be confirmed for sandpipers' nests, but the positive correlation between the predation rate of artificial shorebird nests and incidental observation of arctic foxes (not avian predators) suggests that foxes are likely the main predators of real nests in our study area (unpublished data). Lastly, all nests were considered to be first clutches since our fieldwork starts shortly after snowmelt (i.e., when shorebirds start to lay) and replacement clutches are assumed to be very rare in our study area owing to the very short breeding season.

### 2.4 Predation Pressure

We first calculated the individual predation pressure generated by each fox‐year by estimating the Autocorrelated kernel Utilisation Distribution (UD) of that fox‐year (since our data were correlated in space and time), using the *hr_akde()* function of the *amt* package (Signer et al. 2019). An individual's UD is defined as its intensity of use of an area, based on the probability of having a high or low density of GPS positions (hereafter interpreted as reflecting a high or low predation risk, respectively). Therefore, it describes where the individual has the highest probability of being found (Fortin et al. 2005; Thaker et al. 2011). To allow for unbiased inter‐individual comparisons, we set a grid with a cell size of 50 × 50 m that was identical for all fox‐years. For the fox that had 2 years of tracking, the predation pressure was calculated separately for the different years. The individual UD calculations left us with a raster for each individual fox‐year, with each pixel corresponding to a value of fox UD (with higher values corresponding to a stronger predator presence, that is, a greater assumed predation pressure). UD values were subsequently standardised between 0 and 1 for each fox‐year to allow comparisons of spatial activity between individual foxes (with values close to 1 signalling a high fox activity, thereby a relatively high predation pressure). We then associated to each nest a fox UD value (as a measure of the predation risk it experienced) corresponding to the UD score on the nest location. We only selected the nests that were in at least one studied fox home range in the corresponding study year. Nests that were located outside the territories of tracked foxes were therefore excluded from the analyses, because they were subject to a predation pressure we could not quantify. Only the predation pressure generated during the year the nest was active was considered. As three foxes were monitored at the same site in 2020, we first calculated the cumulative predation pressure experienced by each nest by summing the standardised UD (sUD) values of all individual fox rasters that corresponded to the location of the nest, before dividing this cumulated pressure by the number of foxes used to calculate this value (i.e., the number of foxes whose sUDs' encompassed the bird nest of interest).

### 2.5 Statistical Analyses

#### 2.5.1 Impact of Fox Predation Pressure on Start of Incubation Date

To model start of incubation dates (i.e., the date at which the incubation started) as a function of fox predation pressure, we only kept nest data for which we were able to determine the start of incubation date (*N = 65*). For all models, we included year as a random factor since preliminary analyses showed that the start of incubation dates tended to be clustered by year (Figure S2). We fitted linear mixed models (LMMs) to explain start of incubation dates, using the *lme4* package (Bates et al. 2015). To identify which variables had a significant impact on start of incubation date, we built a full model that included predation pressure (i.e., fox sUD), bird species (dunlin or sanderling), and their interaction. We also built one model for each component of this full model, plus a constant model. We compared these five models using the Akaike information criterion corrected for small sample size (AICc, Burnham et al. 2011; Table 1). We included an interaction between sandpiper species and fox sUD in the full model since predation rates can vary between species (Léandri‐Breton and Bêty 2020). In the AICc comparison procedure, the model yielding the lowest AICc value was considered the best model. In case two or more models were considered equivalent (i.e., ΔAICc < 2; Brown et al. 2022), the model with fewer explanatory variables was considered the best final model (principle of parsimony).

**TABLE 1:** Model selection of the best model explaining start of incubation date in sandpipers. Models were compared using the Akaike information criterion corrected for small sample size (AICc). The model yielding the lowest AICc score was considered the best. In each model, year was included as a random factor to account for inter‐annual variations [(1|year)]. The best final model is indicated in bold. * = Interaction. K = number of parameters estimated. ΔAICc = Difference in AICc score between a given model and the best‐ranked model. AICcwt = AICc model weights. SUD = Standardised fox Utilisation Distribution.

Model	*K*	AICc	ΔAICc	AICcwt
**Start of incubation date ~ Fox sUD × Bird species + (1|year)** *(full model)*	**6**	**414.27**	**0.00**	**0.91**
Start of incubation date ~ Fox SUD+ Bird species + (1|year)	5	419.52	5.25	0.07
Start of incubation date ~ Fox sUD + (1|year)	4	422.49	8.23	0.01
Start of incubation date ~ Bird species + (1|year)	4	424.31	10.04	0.01
Start of incubation date ~1 + (1|year)	3	427.02	12.75	0.00

#### 2.5.2 Impact of Predation Pressure on Daily Survival Rate of Sandpiper Nests

We used Generalised Linear Mixed Models (GLMMs) built with the *lme4* package to test the effect of predation pressure on daily survival rates (DSR; the probability that a nest will survive to the next day within a specific time interval) of dunlins and sanderlings nests. Only nests for which we were able to confidently determine the fate (hatched or predated; *N* = 49 [Table S2.1]) were considered in this analysis. Abandoned nests or nests with an unknown fate were discarded. We used a model accounting for exposure time (i.e., the fact that different nests were discovered at different times and tracked for different durations, Shaffer 2004) and a logistic‐exposure link function (Korner‐Nievergelt et al. 2024) that allows for unbiased DSR estimations since it controls for the different dates of discovery and exposure durations of individual nests. Exposure duration was determined for each nest as the number of days between the date of discovery and the date of hatching or depredation (for successful and predated nests, respectively; Samsonov et al. 2022). The day of discovery was defined as the day when the nest was first discovered. Tracking of individual nests yielded 304 exposure‐days in total (mean = 10; range 1–19 days of exposure per individual nest). We hypothesised that nest DSR was related to fox predation pressure (fox sUD), bird species and date of initiation. However, as start of incubation date was correlated with fox sUD and bird species (see Results), we could not directly include start of incubation date in the model (correlation between two explanatory variables). Instead, we used the residuals of the best model explaining start of incubation date (hereafter, “Residuals”, i.e., the part of variations in start of incubation dates not explained by the predictive variables, see Results) to test for the effect of fox sUD and bird species, controlling for nest initiation date. To account for repeated measures of individual nests and inter‐years variability (see Start of incubation date models), we included Nest ID nested within year as random factor in our models. We worked through the model selection procedure using the AICc and following the same process than for start of incubation date models (see above). We compared eight competing models (Table 2) built according to our hypotheses and biological relevance. Again, we selected the best model using the AICc (see above). Since nest DSR can fluctuate with time and nest age in some bird species (Grabowski et al. 2013; Lei et al. 2021; Que et al. 2015; Weiser et al. 2018a; Zhao et al. 2020), we initially included both variables in preliminary analyses (Table S3.1), but none of them performed better than the constant model (i.e., ΔAICc > 2), so we did not keep them in our final analyses. All analyses were performed using the R software (version 4.3.2; R Core team 2025).

**TABLE 2:** Model selection of the best model explaining nest daily survival rates (DSR) of sandpipers. Models were compared using the Akaike information criterion corrected for small sample size (see details in methods). In each model, nest ID nested within year was included as random factor [(1|year/Nest ID)] to account for repeated measures of individual nests and inter‐annual variations. The best final model is indicated in bold. Residuals = residuals of the best nest initiation model [Start of incubation date ~ Fox sUD*Bird species + (1|year)]. * = Interaction. K = number of parameters estimated. ΔAICc = Difference in AICc score between a given model and the best‐ranked model. AICcwt = AICc model weights. SUD = Standardised fox Utilisation distribution.

Model	*K*	AICc	ΔAICc	AICcwt
**DSR ~ Fox sUD + (1|year/Nest ID)**	**4**	180.34	0.00	0.28
DSR ~ Fox SUD+ Residuals + (1|year/Nest ID)	5	180.59	0.26	0.25
DSR ~ Fox sUD*Bird species + (1|year/Nest ID)	6	180.59	1.25	0.15
DSR ~ Fox SUD+ Bird species + (1|year/Nest ID)	5	182.24	1.91	0.11
DSR ~ Fox sUD*Bird species + Residuals + (1|year/Nest ID) *(full model)*	7	182.64	2.31	0.09
DSR ~ 1 + (1|year/Nest ID)	3	183.46	3.12	0.06
DSR ~ Residuals + (1|year/Nest ID)	4	183.95	3.62	0.05
DSR ~ Bird species + (1|year/Nest ID)	4	185.17	4.83	0.02

## 3 Results

### 3.1 Nest Initiation

**FIGURE 2:**
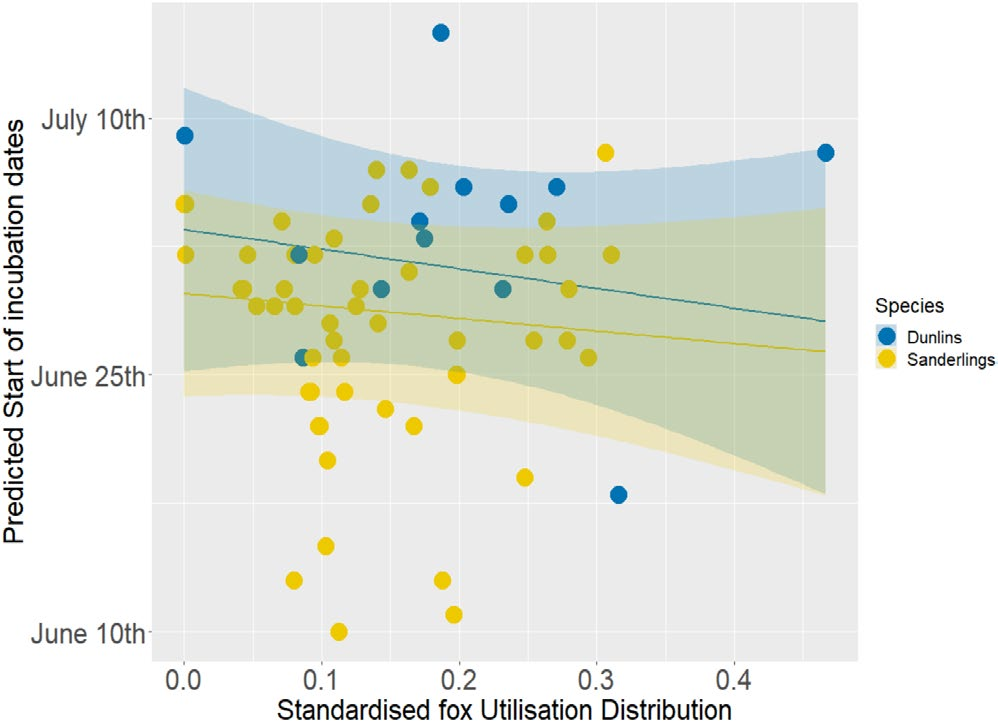
Start of incubation dates of sandpipers in Northeast Greenland according to Standardised fox Utilisation Distribution (sUD) and bird species. High sUD values depict higher fox activity, hence higher predation risk. The lines represent the relationship predicted by the best model, and shaded areas represent estimated 95% confidence intervals. Dots represent observed start of incubation dates.

The sUDs values associated with shorebird nests were between 10^−6^ and 0.47 (mean = 0.16). The best model explaining the start of incubation date in sandpipers contained fox sUD, bird species, and their interaction (Table 1 and Table S4.1). The two species showed differences in their nesting phenology, with dunlins initiating their nests later than sanderlings: all years combined, mean start of incubation date was 3rd of July (range: 18th of June–15th of July) for dunlins and 28th of June (10th of June‐8th of July) for sanderlings. Nests were initiated earlier in areas with higher fox sUD values (i.e., riskier areas) for both species (Figure 2). The interaction between fox sUD and bird species was retained in the best model (Table 1 and Table S4.1). This emphasises a species‐specific response to predation risk, with a slightly larger effect of predation pressure on dunlins (i.e., larger difference in start of incubation dates between low and high‐risk areas; Figure 2). All nests were located in areas associated with a relatively low fox sUD (mean ± SD: 0.15 ± 0.09; maximum: 0.47). Only one nest, which was not an error of reporting, was associated with a fox sUD superior to 0.32.

### 3.2 Daily Survival Rates

**FIGURE 3:**
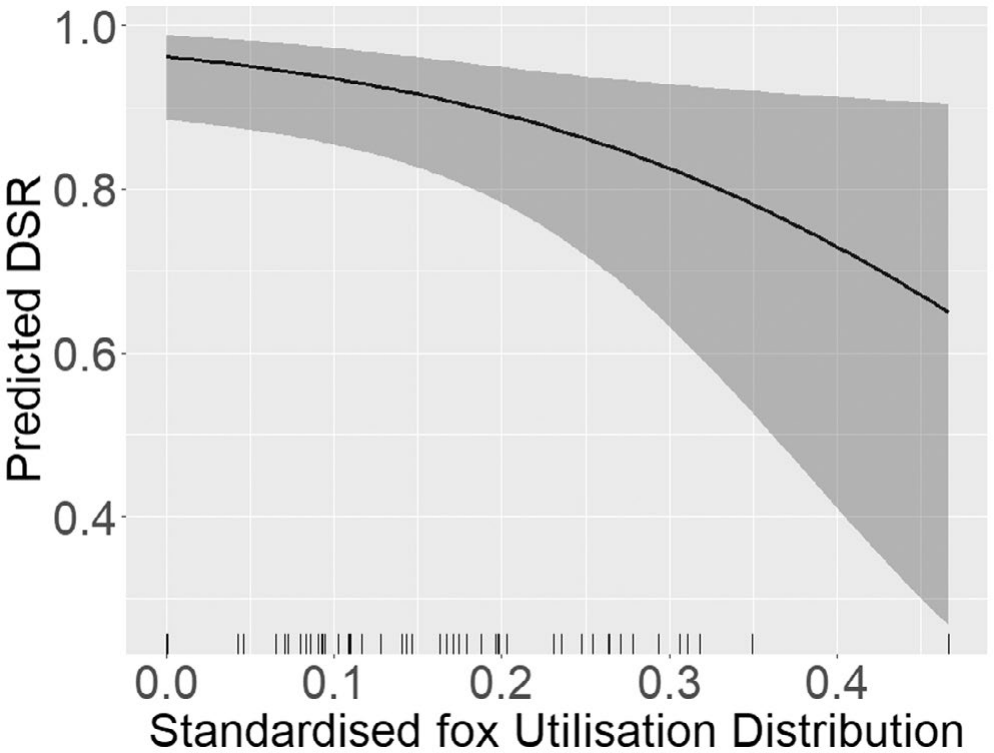
Predicted daily survival rates (DSR) of sandpiper nests in Northeast Greenland according to standardised fox Utilisation Distribution (sUD). High sUD values depict higher fox activity, hence higher predation risk. The lines represent the relationship predicted by the best model, and shaded areas represent estimated 95% confidence intervals. Ticks on the x‐axis correspond to the fox sUD observed for each sandpiper nest in the study.

Among the 49 nests included in our analyses, 27 (55.1%) were depredated (Table S4.2). The mean nest DSR value was 0.915 ± 0.022 for both species combined (mean ± SD; range 0.872–0.948), meaning that only 14% (i.e., 0.915 ^22^) of all the nests would survive over the 22 days incubation period and produce youngs. According to the model selection procedure, variations in nest DSR were best explained by fox sUD only (Table 2 and Data [Link], [Link]), with higher predation pressure (i.e., higher sUD values) associated with lower DSR (Figure 3 and Table S4.2).

## 4 Discussion

We investigated the impact of predation risk by arctic foxes on the breeding behaviour of two species of sandpipers, their incidental prey. According to our second prediction, we found that daily survival rates of sandpiper nests were lower in riskier areas. However, sandpiper nests were initiated earlier in riskier areas (i.e., where arctic foxes have a greater probability of being found), which contradicts our first prediction, and this pattern was slightly modulated by bird species. Together, these results show that arctic foxes exert a strong predation pressure on sandpiper nests, but that sandpipers are probably constrained by larger scale environmental and ecological processes in their nesting phenology.

### 4.1 Earlier Nest Initiation in Riskier Areas

Although the arrival of sandpipers on their Arctic breeding ground is often highly synchronised, variability still remains in start of incubation dates (Leung et al. 2018; Nightingale et al. 2024; Smith et al. 2010). Nesting early can provide strong advantages for birds in the Arctic. Some studies suggest that nests initiated earlier have higher survival rates (Claassen et al. 2014; Grant et al. 2005; Harris et al. 2005; McGuire et al. 2020; Weiser et al. 2018a; but see Reneerkens et al. 2016; Smith and Wilson 2010), probably owing to favourable weather conditions (Plaschke et al. 2019; Van De Pol et al. 2010), to a “dilution effect” of predation (Gorosito et al. 2024; Reneerkens et al. 2016), and/or because of individual characteristics (e.g., older individuals with higher breeding success arriving or breeding earlier; Leyrer et al. 2012; Oring and Lank 1982). Initiating a nest early also gives the opportunity to lay a replacement clutch if needed (Claassen et al. 2014; Gates et al. 2013; Saalfeld et al. 2021) and increases the hatching success of such second clutches (Saalfeld et al. 2021), which apparently constrains birds more than waiting for safer nesting opportunities later in the season.

Our analyses did not support such a positive relationship (i.e., earlier nest initiation in safer, and later in riskier areas) in our study area and our first prediction had to be rejected. The higher predation pressure associated with earlier clutch must hence be a consequence of larger‐scale environmental processes driving nesting phenology in sandpipers. In the high‐Arctic, early breeding birds have a limited choice of nesting sites since snow‐free breeding areas can be scarce at the beginning of the summer (Liebezeit et al. 2014; Meltofte 2007). Weather and snow conditions have also proven to be important drivers of nesting phenology in Arctic‐breeding birds (English et al. 2025; Freeman et al. 2023; Grabowski et al. 2013), sometimes of greater importance than predation risk (Liebezeit et al. 2014; Smith et al. 2010). Since arctic foxes also preferentially forage in the first snow‐free patches at this period, higher predation pressures can be found in these areas (Byrkjedal 1980; Machín et al. 2019; Smith et al. 2010). The rejection of our first prediction could hence also be seen as an indirect support of this “snowmelt hypothesis”. Quantifying snowmelt patterns at the microscale and linking them to predation risk early in the season could help us understand if predators preferentially use snow‐free patches early in the season, resulting in an extreme predation risk in these areas early in the season.

Interestingly, all sandpiper nests in our study were in areas of relatively low predation risk (i.e., sUD values < 0.5), which suggests that sandpipers were either able to actively select areas of lower predation risk (Clermont, Grenier‐Potvin, et al. 2021; Lamarre et al. 2017) or that nests in high‐risk areas were depredated early in the season. The relationship between fox sUD and start of incubation dates was mainly driven by the nest associated with the highest fox sUD, but the results we observed would probably be confirmed if more nests had been discovered in risky areas.

Other ecological drivers may also play a role in triggering start of incubation dates in our model species. For example, sandpipers' nesting phenology is tightly linked to that of arthropods emergence (Lameris et al. 2022; Leung et al. 2018), with hatching time ideally synchronised with the peak abundance of arthropods. As chicks' body condition and survival greatly depend on this seasonally abundant but relatively ephemerous food source (Lameris et al. 2022; McKinnon et al. 2012; Saalfeld et al. 2021), the selection for earlier nest initiations (i.e., to better match with the peak abundance of arthropods) would hence be stronger than those imposed by predation pressure in our study area (Meltofte et al. 2007). These contrasting relationships between the start of incubation date and predation pressure likely reflect the different strategies used by arctic sandpipers to balance between the costs and benefits of initiating their nests early to optimise nesting success.

### 4.2 Impact of Predation on Daily Survival Rates

The daily nest survival rates we found were slightly lower than the nest DSR of other shorebird or similar‐sized species in other Arctic and temperate regions (being generally higher than 0.92; Claassen et al. 2014; English et al. 2017; McFarland et al. 2017; Que et al. 2015; Weiser et al. 2018b). DSR of shorebird nests can greatly vary between years and regions, owing to climatic variations or extreme weather events (Meltofte 2007; Van Irsel et al. 2022; Weiser et al. 2018b). However, differences in predator guild composition and abundance are also likely to play a crucial role, since predators other than the arctic fox can also exert a strong predation pressure on breeding shorebirds (Bentzen et al. 2017; Brown et al. 2022). Although the predator guild is relatively limited in our study area, the respective contribution of each predator to the total predation rate is currently unknown. However, as arctic foxes are the most abundant predators in the area, they are probably also responsible for most depredation events. Our study system is comparatively poor in terms of food resources. Previous studies have shown that birds and their eggs represent a significant part of arctic foxes' diet in Northeast Greenland, especially in times of food scarcity (i.e., lemming crashes; Schmidt et al. 2022). As lemming population dynamics are dampening in the Arctic (Gilg et al. 2009; Kausrud et al. 2008), with shorter and scarcer periods of peak abundance, nest survival could therefore experience cascading impacts in the future.

Our results showed a negative effect of fox predation pressure on the DSR of sandpiper nests, corroborating the findings of other studies (Flemming, Nol, Kennedy, Bédard, et al. 2019; Léandri‐Breton and Bêty 2020; McKinnon et al. 2014). Sandpipers and other shorebirds are considered incidental prey for arctic foxes throughout their range (Cornell 1976; Mckinnon, Berteaux, et al. 2013; McKinnon, Nol, and Juillet 2013). However, they can still represent a significant proportion of arctic foxes' diet in Northeast Greenland (Schmidt et al. 2022), and foxes are usually the main predators of shorebird nests in the Arctic (Liebezeit and Zack 2008; McKinnon and Bêty 2009). It is generally assumed that arctic foxes do not actively search for shorebird nests, preferring larger or more nutritious prey such as geese and lemmings (Beardsell et al. 2023; Dalerum and Angerbjörn 2000; Dulude‐de Broin et al. 2023; Schmidt et al. 2022). However, an increase in movements from and to specific hunting areas where preferred prey aggregate could result in the accidental discovery and consumption of more shorebird nests (Dulude‐de Broin et al. 2023), showing an indirect relationship between arctic foxes' movements and shorebirds' breeding ecology. Despite their crypticity (Mayer et al. 2009; Skrade and Dinsmore 2013), shorebird nests are maybe more easily detected by foraging foxes than we think, especially if they are located in preferred hunting areas (Flemming, Nol, Kennedy, Bédard, et al. 2019; Flemming et al. 2016; Lamarre et al. 2017). Nests are also possibly more prone to predation if they are located on ridges or relatively dry areas that are used by foxes as denning sites or for travelling (Johnson‐Bice et al. 2024; Zhao et al. 2020). A finer‐scale description of prey distribution and landscape characteristics would help to better map predation risk and assess its consequences at smaller spatial scales.

Our results corroborate those obtained with artificial nests elsewhere (e.g., Brown et al. 2022; Flemming, Nol, Kennedy, Bédard, et al. 2019; Lamarre et al. 2017; Lecomte et al. 2008; Mckinnon, Berteaux, et al. 2013; McKinnon, Nol, and Juillet 2013). However, studies using artificial nests can potentially be biased since such nests do not present the biological and behavioural characteristics of incubating birds. This could explain why we generally found higher global survival on our real nests compared with artificial nests. For instance, Brown et al. (2022) found that only about 20% of nests survived the 10‐day period. Several studies have emphasised that movements and parental care strategies of incubating shorebirds influence predation rates (Meyer et al. 2020; Smith et al. 2012). As such, using artificial nests to assess the impact of predation risk on breeding birds could lead to erroneous or at least incomplete conclusions (Major and Kendal 1996; Moore and Robinson 2004). In addition, most studies on the interactions between shorebirds and arctic foxes have been carried out in highly productive “hotspots” (i.e., areas with a high density of preferred prey such as geese) with relatively higher nest survival rates (Beardsell et al. 2023; Dulude‐de Broin et al. 2023; Flemming, Nol, Kennedy, Bédard, et al. 2019; Flemming et al. 2016), which also calls into question their representativeness for High Arctic ecosystems, the vast majority of which are low‐productivity systems. Our study is thereby one of the few to describe the impact of arctic foxes' predation pressure on breeding sandpiper (i.e., incidental prey) in a representative (i.e., relatively poor) high‐Arctic study area.

### 4.3 Temporal and Species‐Specific Trends

Interestingly, we did not find an effect of time nor nest age on DSR of sandpiper nests in our study. This contrasts with results from other Arctic studies that found strong temporal variations in nest DSR during the breeding season (e.g., Grabowski et al. 2013; Smith and Wilson 2010; Weiser et al. 2018a). These temporal trends were at least partly attributed to changes in predator foraging behaviour following the availability of alternative prey over time (Smith and Wilson 2010; Weiser et al. 2018a). However, in the case of a relatively stable availability of preferred prey, one should expect no or less pronounced temporal trend in nest depredation. Our study area presents a relatively poor but rather diversified prey assemblage for arctic foxes that feed on a variety of different prey items over the summer (Schmidt et al. 2022). Additionally, lemming cycles have recently dampened in Northeast Greenland as a result of climate change (Gilg et al. 2009), resulting in less pronounced inter‐annual variation in prey availability. Thereby, the quantity of prey available to arctic foxes might stay relatively stable throughout the summer (e.g., with no strong summer decline of lemming densities as experienced during peak years; Gilg 2002), explaining the absence of strong temporal variations in our study system.

We identified species‐specific responses to arctic fox predation risk in terms of nest initiation, with dunlins reacting slightly more strongly to predation pressure. Interspecific differences in terms of habitat preferences, tolerance to environmental conditions, and foraging ecology could potentially explain this pattern (Cunningham et al. 2016; Mckinnon, Berteaux, et al. 2013; McKinnon, Nol, and Juillet 2013; Meltofte 2007). Dunlins preferentially nest in wet (fen) tundra (Cunningham et al. 2016) whereas sanderlings nest in areas of relatively low normalised difference vegetation index (NDVI) and avoid wet habitats (Pellissier et al. 2013). Species‐specific differences in breeding phenology are therefore most likely explained by different micro‐climatic conditions found in their respective breeding habitats. Indeed, breeding habitats of dunlins and sanderlings differ in terms of moisture, vegetation cover and temperature at the micro‐scale (Von Oppen et al. 2022). Finally, because they were potentially less constrained in their nest initiation patterns, sanderlings could have reacted less strongly to predation risk, their nesting phenology being driven by the availability of nesting sites in particular habitat types (e.g., available more or less early depending on snow and weather conditions), by a stronger sensibility to trophic mismatch (e.g., considering that arthropods are generally less abundant in their drier breeding habitats, Bolduc et al. 2013) or a higher tolerance to predation risk overall. Regarding DSR, previous studies found that habitat selection patterns can influence nest predation rates in shorebirds (Léandri‐Breton and Bêty 2020), but our sample size was possibly too small to detect species‐specific DSR. Recording nest habitats of a large number of sandpipers would then allow us to test this hypothesis in our study area.

## 5 Limitations

Due to field and logistic constraints, we were unable to fit all foxes present in our study areas with GPS collars. Although they can be considered conservative (i.e., obtained with only a fraction of the real fox population), our estimates of predation pressure were not perfectly representing the true predator activity in the area (Clermont, Grenier‐Potvin, et al. 2021). However, since we divided the total sUD obtained by the number of foxes that generated it, our predation pressure index partially controlled for this bias. Arctic foxes being generally very territorial, especially when resources are relatively scarce (such as in our study area; Follmann and Fay 1982; Goltsman et al. 2005; Pletenev et al. 2021), the presence of “floaters” (i.e., foxes without a stable home range; Beardsell et al. 2023), dispersing individuals, or neighbouring foxes probably contributed little to the predation pressure exerted by foxes on breeding sandpipers. As such, we assume that our measures of predator activity correctly depict the predation pressure experienced by sandpipers in a given area. Some foxes were also followed for relatively small periods, but similar studies used comparable tracking durations (Clermont, Woodward‐Gagné, and Berteaux 2021; Poulin et al. 2021).

## 6 Conclusion

We showed that arctic foxes impose a significant predation pressure on Arctic‐breeding sandpipers in Northeast Greenland, with lower daily survival rates and earlier start of incubation dates for nests located in areas of high fox activity. Our results give an insight into the impact of predation risk by arctic foxes on the breeding success and phenology of Arctic‐breeding sandpipers using real nests data. This contributes to a better understanding of predator–prey interactions in the fast‐changing Arctic ecosystems, particularly exposed to ongoing climate change and the resulting shifts in predator–prey interactions (Gilg et al. 2012, 2009; Kubelka et al. 2022; Schmidt et al. 2017).

## Author Contributions

**Laura Bonnefond:** conceptualization (lead), formal analysis (lead), visualization (lead), writing – original draft (lead), writing – review and editing (lead). **David Pinaud:** conceptualization (equal), formal analysis (equal), methodology (equal), supervision (lead), validation (equal), visualization (equal), writing – review and editing (lead). **Loïc Bollache:** funding acquisition (lead), project administration (lead), validation (equal). **Thomas Pagnon:** data curation (equal), resources (equal), validation (equal), writing – review and editing (equal). **Eric Buchel:** data curation (equal), resources (equal), validation (equal). **Brigitte Sabard:** data curation (equal), resources (equal), validation (equal). **Vladimir Gilg:** data curation (equal), resources (equal), validation (equal). **Jérôme Moreau:** conceptualization (equal), funding acquisition (lead), methodology (equal), project administration (equal), supervision (lead), validation (equal), writing – review and editing (lead). **Olivier Gilg:** conceptualization (equal), data curation (lead), funding acquisition (equal), methodology (equal), project administration (lead), resources (lead), supervision (lead), validation (equal), writing – review and editing (lead).

## Funding

This study is part of the long‐term Studies in Ecology and Evolution (SEE‐Life) program of the CNRS and was funded by the French Polar Institute‐IPEV (“Interactions 1036” Program), the Agence Nationale de la Recherche (ANR‐21‐CE02‐0024 PACS), the Groupe de Recherche en Ecologie Arctique (GREA), the University of Bourgogne Europe and the University Marie et Louis Pasteur.

## Conflicts of Interest

The authors declare no conflicts of interest.

## Supporting information


**Data S1:** Supporting information.

**Data S2:** Supporting information.

**Data S3:** Supporting information.

**Data S4:** Supporting information.

## Data Availability

All data used and analysed during this study are accessible on Movebank (https://www.movebank.org/cms/webapp?gwt_fragment=page=studies,path=study1255899828; https://www.movebank.org/cms/webapp?gwt_fragment=page=studies,path=study1255911964; https://www.movebank.org/cms/webapp?gwt_fragment=page=studies,path=study1255889609) and are available from the corresponding author upon request. The .csv data files and the code used to prepare and analyse the data are available as Data [Link], [Link].
