# Supplementary material for: Predation Risk Does Not Delay Breeding but Reduces Nest Survival in High‐Arctic Shorebirds
Source: Ecol Evol. 2026 Mar 18;16(3):e72997. doi: 10.1002/ece3.72997 (PMC13093658; doi:10.1002/ece3.72997)
Supplement: Supplementary file 1 — Data S1: Supporting information. [file ECE3-16-e72997-s002.docx]

**Supplemental Information for:**

**Predation Risk Does Not Delay Breeding but Reduces Nest Survival in High-Arctic Shorebirds**

**Table of Contents:**

| **Sandpiper nests and fox data summary** | **Page 2** |
| --- | --- |
| **Yearly structure of start of incubation dates and nest fate data** | **Page 3** |
| **Model selection of daily survival rate models according to**  **nest age and Julian day** | **Page 4** |
| **Summary tables of the best models explaining start of incubation dates**  **and daily survival rates of sandpiper nests** | **Page 5** |

**Appendix S1. Sandpiper nests and fox data summary.**

Table S1.1: Summary table of sandpiper nesting periods in our study area. Dates are written as Year-Month-Day. The duration of this period is defined by the difference between the earliest start of incubation date (determined by floating eggs from the nest upon discovery; see Methods) and the latest date of hatching (determined by Tiny Tag data and/or direct observations). Only fox data collected during the nesting period in each year were kept to estimate predation risk.

|  | 2017 | | 2020 | | 2021 |
| --- | --- | --- | --- | --- | --- |
| First nest discovery | 2017-07-12 | 2020-07-03 | | | 2021-07-02 |
| Earliest start of incubation | 2017-06-26 | 2020-06-17 | | | 2021-06-10 |
| Latest hatching | 2017-08-06 | 2020-07-28 | | | 2021-07-27 |
| Duration | 41 | 41 | | 47 | |

Table S1.2: Summary of the summer GPS data obtained after data filtering post-cleaning (see Methods) for the five fox-years used in this study. Information for the fox that was tracked for two consecutive years (yielding two fox-years of data) is indicated in italics. The number of tracking days indicates the number of days for which the given fox was tracked during the sandpipers’ breeding season.

| Sex | Fox ID | Number of GPS positions | First day of tracking (Y-M-D) | Last day of tracking (Y-M-D) | Number of tracking days |
| --- | --- | --- | --- | --- | --- |
| Female | 928001 2017 | 2,185 | 2017/07/22 | 2017/08/02 | 11 |
|  | 928029 2020 | 1,140 | 2020/07/25 | 2020/07/28 | 4 |
| Male | *928031 2020* | *1,271* | *2020/07/25* | *2020/07/28* | *4* |
|  | *928031 2021* | *13,873* | *2021/06/10* | *2021/07/27* | *48* |
|  | 928048 2020 | 3,751 | 2020/07/18 | 2020/07/28 | 11 |

**Appendix S2. Yearly structure of start of incubation dates and nest fate data.**


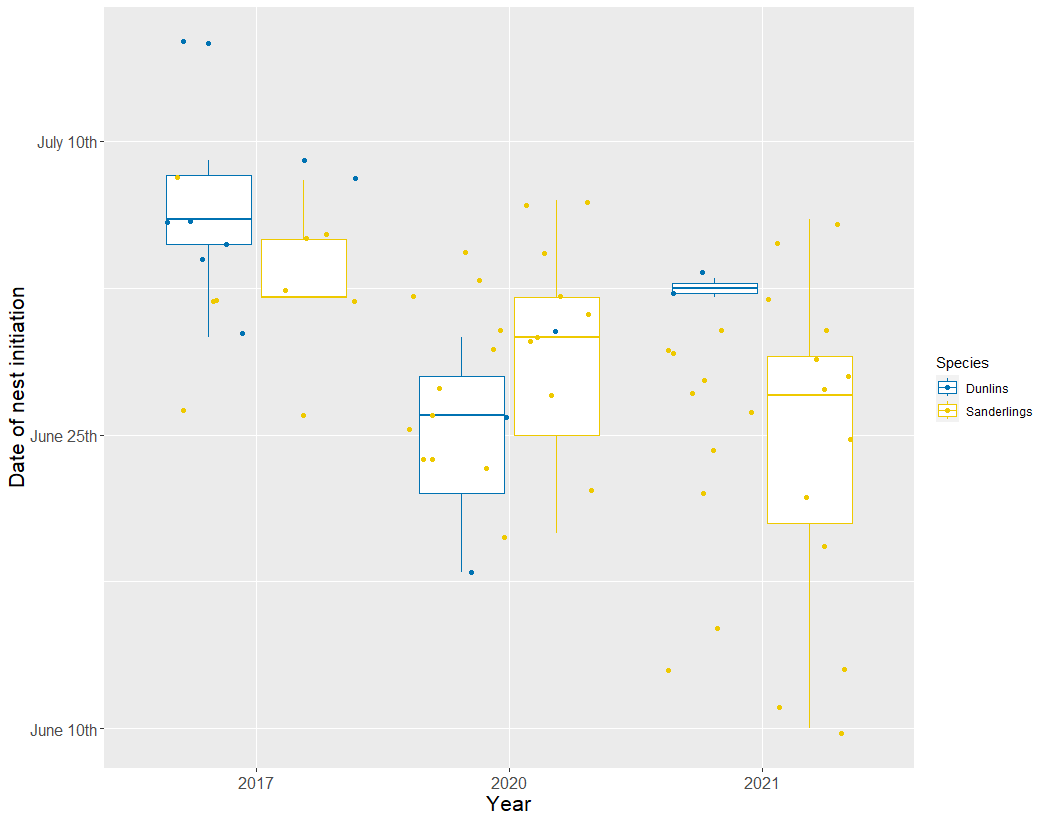


Fig. S2.1: Observed start of incubation dates of sandpipers in Northeast Greenland according to year of study and bird species. We only considered nests located inside a fox territory.

Table. S2.1: Nest fate of sandpipers in Northeast Greenland according to year of study and bird species [dunlins (*Calidris alpina arctica*) and sanderlings (*Calidris alba*)]. Nest fate was determined based on Tiny Tags data or direct observations (see Methods). Values indicate the number of predated nests/total number of nests tracked for this species during the considered year.

| Species | 2017 | 2020 | 2021 |
| --- | --- | --- | --- |
| Dunlins | 5/7 | 0/2 | 2/2 |
| Sanderlings | 5/7 | 12/15 | 3/16 |

**Appendix S3. Model selection of daily survival rate models according to nest age and Julian day.**

Table. S3.1: Model selection of the best model explaining nest daily survival rates (DSR) by Nest age and Julian day (temporal variations). Julian day was included as both linear and quadratic [ (Julian Day)^2 ] predictor. Models were compared using the Akaike information criterion corrected for small sample size (AICc; see Methods). In each model, nest ID nested within year was included as random factor to account for repeated measures of individual nests and inter-year variations. The best model is indicated in bold. K = number of parameters estimated. ΔAICc = Difference in AICc score between a given model and the best-ranked model. AICcwt = AICc model weights. sUD = Standardised fox Utilisation distribution.

| Model | *K* | AICc | ΔAICc | AICcwt |
| --- | --- | --- | --- | --- |
| **DSR ~ Fox sUD + (1\|year/ Nest ID)** | **4** | **180.34** | **0.00** | **0.67** |
| DSR ~ 1 + (1\|year/ Nest ID) | 3 | 183.46 | 3.12 | 0.14 |
| DSR ~ Nest age + (1\|year/ Nest ID) | 4 | 184.86 | 4.52 | 0.07 |
| DSR ~ Julian Day + (1\|year/ Nest ID) | 4 | 185.17 | 4.83 | 0.06 |
| DSR ~ (Julian Day)^2 + (1\|year/ Nest ID) | 4 | 185.17 | 4.83 | 0.06 |

**Appendix S4. Summary tables of the best models explaining start of incubation dates and daily survival rates of sandpiper nests.**

Table S4.1: Summary table of the best model explaining start of incubation dates of sandpipers according to standardised fox Utilisation Distribution (sUD), bird species, and their interaction. Year was included as random factor to account for inter-year variations. Reference category for bird species = dunlins. * = interaction.

|  | Estimate ± standard error | *t* value |
| --- | --- | --- |
| Intercept | 184.50 ± 4.23 | 43.61 |
| Fox sUD | -11.49 ± 15.11 | -0.76 |
| Bird species (Sanderling) | -3.74 ± 3.84 | -0.97 |
| Fox sUD*Bird species | 4.17 ± 18.15 | 0.23 |

Table S4.2: Summary table of the best model explaining daily survival rates of sandpiper nests according to standardised fox Utilisation Distribution (sUD). Nest ID nested within year was included as random factor to account for repeated measures of individual nests and inter-year variations.

|  | Estimate ± standard error | Z value | *p-value* |
| --- | --- | --- | --- |
| Intercept | 3.23 ± 0.61 | 5.33 | 9.89e-08 |
| Fox sUD | -5.60 ± 2.47 | -2.27 | 0.0231 |
